# Effects of curing modes on depth of cure and microtensile bond strength of bulk fill composites to dentin

**DOI:** 10.1590/1678-7757-2019-0753

**Published:** 2020-07-03

**Authors:** Sara N. MAKHDOOM, Karen M. CAMPBELL, Ricardo Marins CARVALHO, Adriana Pigozzo MANSO

**Affiliations:** 1 The University of British Columbia Faculty of Dentistry Department of Oral Health Sciences Vancouver Canada The University of British Columbia, Faculty of Dentistry, Department of Oral Health Sciences, Vancouver, Canada.; 2 University of Toronto Faculty of Dentistry Graduate Program in Pediatric Dentistry Toronto Canada University of Toronto, Faculty of Dentistry, Graduate Program in Pediatric Dentistry, Toronto, Canada.; 3 The University of British Columbia Faculty of Dentistry Department of Oral Biological and Medical Sciences Vancouver Canada The University of British Columbia, Faculty of Dentistry, Department of Oral Biological and Medical Sciences, Vancouver, Canada.

**Keywords:** Bulk fill, Depth of cure, Bond strength, Light curing

## Abstract

**Objectives:**

To compare the microtensile bond strength (µTBS) and depth of cure (DOC) of bulk-fill composites cured by monowave (MW) and polywave (PW) LED units using different curing times.

**Methodology:**

Three composites were tested: Tetric EvoCeram Bulk Fill (TBF), Filtek Bulk Fill (FBF), and Tetric EvoCeram (T; control). Flat dentin surfaces treated with adhesive (AdheSE Universal^®^, Ivoclar Vivadent) were bonded with 4 mm cylindrical samples of each bulk-fill composite material (n=6) and cured with monowave (Satelec) or polywave (Bluephase Style) curing units for 10 or 20 seconds. After 24 hours, teeth were sectioned into individual 0.9 mm^2^ beams and tested for µTBS. Failure modes were analysed. Moreover, the DOC scrape test (IOS 4090) was completed (n=5) following the same curing protocols. Two-way ANOVA (a=0.05) was performed, isolating light-curing units.

**Results:**

For samples cured with the MW light-curing unit, no significant effects were observed in the µTBS results between any of the resin composite brands and the curing times. Conversely, when resins were cured with a PW light unit, a significant effect was observed for TBF resin. In general, bulk-fill composites presented greater DOC and longer curing time resulted in higher DOC for all composites.

**Conclusion:**

The µTBS of the composites to dentin was not affected by the curing mode of the resins, except for TBF cured with PW light unit. Bulk-fill composites exhibit greater DOC than conventional resin-based composites.

## Introduction

With the trend moving away from the use of amalgam restorations, the demand for resin-based direct restorative materials has increased.^1^ Their bonding capability, relative stability, and acceptable clinical performance in the oral environment makes these materials well-suited to minimally invasive restorative procedures. Resin composite development has evolved, with changes in fillers, monomers, and/or curing systems.^2^ The recent introduction of bulk-fill resin composites aimed to streamline the clinical application of resin composites by accommodating curing in 4-5 mm increments. Bulk-fill composites have been designed to enhance light transmittance and depth of cure (DOC) compared to conventional resin composites.^3,4^Although these modifications vary, most manufacturers aim for a more translucent material with enhanced curing capability through filler modifications,^2,3^ incorporation of high molecular weight monomers, and/or addition of new alternative photoinitiators.^2,5-8^ Several commercially available bulk-fill materials have increased filler size or decreased filler content to minimize the scattering of light, thus encouraging light transmittance.^2,7^ Moreover, modifications to monomers and photoinitiator targets improved optical properties, reduced polymerization shrinkage, and increased DOC.^8-12^

Bulk-fill composites are commercially available in different viscosities, with different clinical applications; however, the impact of those modifications on performance is not yet fully understood. In addition to camphorquinone in bulk-fill composites, the use of alternative photoinitiator systems has been reported as the main factor contributing to enhanced DOC.^12^ Alternative photoinitiators such as Ivocerin, Irgacure 819, and OPPI (onium compound p-octyloxy-phenyl-phenyl-iodonium hexafluoroantimonate) have absorption peak wavelengths ranging from 290 to 330 nm, which does not match with monowave (MW) or single-peak light-emitting diode (LED) curing units (ranging from 350 to 460 nm).^13^This mismatch identifies a parallel concern related to whether single-peak LED light-curing units can efficiently cure bulk-fill composites containing alternative photoinitiators.^14,15^However, research on the impact of single-peak LED curing units on the performance of bulk-fill composites is still scarce.^16^

Several independent studies have validated the DOC on bulk-fill composites with the use of different light-curing units,^17-19^and the degree of conversion of bulk-fill materials.^4,11,14,16,20-24^ Although a few studies have investigated shear bond strength (SBS)^25^ and the μTBS of bulk-fill composites compared to conventional ones,^26,27^ none have explored the impact of different LED curing units and curing times on the resin-dentin bond strengths and DOC of restorative bulk-fill resin composites. Our study sought to evaluate the effects of polywave (PW) and monowave (MW) LED curing units on the microtensile bond strength (µTBS) of the composites to dentin and on the depth of cure (DOC) of restorative bulk-fill resin composites using two different curing times.

## Methodology

**Figure 1:**
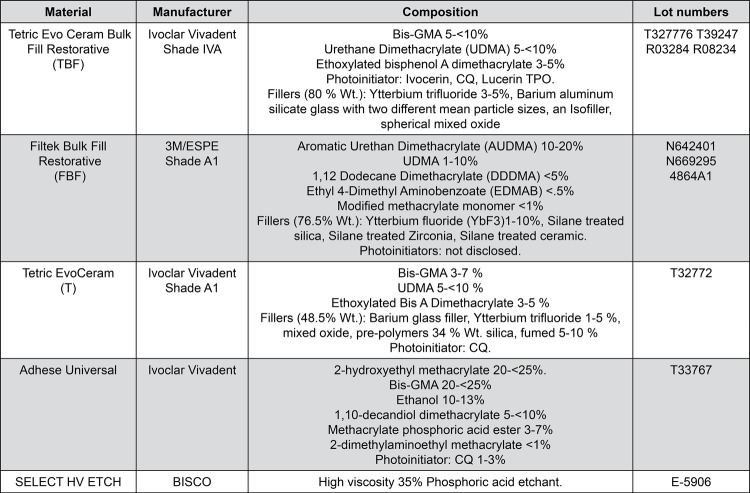
List of materials, manufacturers, composition and lot numbers

Two commercially available restorative (i.e., non-flowable) bulk-fill composites, Tetric EvoCeram Bulk-Fill restorative (TBF; Ivoclar Vivadent, Schaan, Liechtenstein) and Filtek Bulk-Fill restorative (FBF; 3M/ESPE, St Paul, MN, USA), and one conventional restorative resin composite, Tetric EvoCeram (T; Ivoclar Vivadent, Schaan, Liechtenstein), were used in our study. A single adhesive system AdheSE Universal^®^ (Ivoclar Vivadent, Schaan, Liechtenstein) was used for all groups, following the same bonding protocol (Figure 1), as described below. The light-curing units used in our study were: Monowave (MW) Light Emitting Diode – Satelec MiniLED Supercharged (SATELEC^®^, ACTEON^®^, Mérignac, Bordeaux, France) and Polywave (PW) Light Emitting Diode – Bluephase Style^®^ (Ivoclar Vivadent, Schaan, Liechtenstein). The tips of the two light-curing units were both 7.5 mm in diameter to ensure that the expected radiance was equally delivered to all specimens. The light output of each unit was monitored daily using their corresponding radiometers: Satelec MiniLED Supercharged radiometer (SATELEC^®^, ACTEON^®^, Mérignac, Bordeaux, France), or Bluephase Meter II radiometer (Ivoclar Vivadent, Schaan, Liechtenstein). Curing times were either 10 or 20 seconds. The irradiance energy for both curing units was set at ≅ 960 mW/cm^2^ with a total energy of 9.6 J/cm^2^at 10 seconds and 19.2 J/cm^2^at 20 seconds.

### Microtensile bond strength (µTBS) test

Seventy-two extracted, non-carious third molars were collected and stored in 0.1% thymol solution at 4°C. The teeth were wet polished (Precision Lapping/Polishing machine, MTI Corporation, EQ-UNIPOL-1210, Richmond, BC, Canada) with 180-grit silicon carbide (SiC) paper to expose flat dentin surfaces, parallel to the cemento-enamel junction (CEJ). Before the dentin bonding procedure, each dentin surface was manually polished with 320-grit SiC paper (Norton, Worcester, MA, USA) for 10 seconds to create a standardized smear layer. Dentin surfaces were etched with 35% phosphoric acid (Select HV Etch, BISCO, Schaumburg, IL, USA) for 15 seconds and thoroughly rinsed with water for 15 seconds. The demineralized dentin surface was blot dried with filter paper to achieve uniform surface moisture before adhesive application. A multimode adhesive, AdheSE Universal^®^ (Ivoclar Vivadent, Schaan, Liechtenstein), was actively applied using a rubbing motion for 20 seconds and the remaining solvent was evaporated by air circulation for 10 seconds. The adhesive was light-cured using the corresponding curing unit for 10 seconds at 960 mW/cm^2^. This approach was selected to prevent a potential confounding factor of using a different light curing unit for the adhesive layer and the subsequent composite layer.

Cylindrical matrices, measuring 4 mm in height and 7.5 mm in diameter (to match the diameter of both light-curing unit tips), were created with polyvinyl siloxane (Aquasil Ultra LV/XLV Smart Wetting^®^ Regular Set, Dentsply) to support the bulk of the resin composite while applied to the bonded dentin surface. Each resin composite tested was cured in 4 mm bulk-filled increments, under one of the following four different curing protocols: using MW (Satelec MiniLED Supercharged, SATELEC^®^,ACTEON^®^, Mérignac, Bordeaux, France) for either 10 or 20 seconds; or PW (Bluephase Style^®^, Ivoclar Vivadent, Schaan, Liechtenstein) for either 10 or 20 seconds. After light curing, the matrix was removed and each specimen was immediately stored in distilled water in a dark incubator at 37°C.

After 24-hour storage, the specimens were sliced into approximately 0.9 mm^2^beams using a slow speed diamond saw at 300 rpm (SYJ-150 slow speed diamond saw, MTI Corporation, SYJ-150 Richmond, BC, Canada). Each beam was individually measured at the interface with a digital caliper to the nearest 0.01 mm (Fisher Scientific, Chicago, IL, USA) before testing. For the µTBS tests, each beam was stabilized on metallic slabs (Odeme Dental Research, Joaçaba, SC. Brazil) using cyanoacrylate glue, then mounted in a Universal Testing Machine (SHIMADZU Corporation, AutoGraph AGS-X, Kyoto, Japan). Tensile force was applied at a crosshead speed of 0.5 mm/min. Final µTBS values were expressed in MPa. The bond failure modes were evaluated under 20× magnification using a Leica MZ6 (Leica Microsystems Inc, Concord, ON, Canada) optical microscope. Failure modes were classified as cohesive, adhesive, or mixed. Representative beams of each failure mode were mounted on aluminum stubs and sputter coated with 20 nm Iridium (Leica EM MED020, Leica Microsystems Inc, Concord, ON, Canada) for imaging with a scanning electron microscope (Hitachi SU-3500, Hitachi High Technologies, Rexdale, Ontario, Canada).

### Depth of cure (DOC) scrape test

Individual unit-dose capsules of each resin composite were used as molds for the scrape test, as described by ISO 4049 specifications.^28^ The capsule back-end and nozzle-end were removed and a 12 mm capsule cylinder remained. The resin composite inside each capsule was condensed against a Mylar strip on a glass slab and then cured using the same protocols described previously for the µTBS test. The uncured resin composite was immediately scraped off from the ejected cylinders with a metallic spatula (765 Premium Instrument, AISI 420, Germany). What remained of the resin composite cylinder after the scraping procedure was measured and divided by two to determine the DOC in millimeters.

Additional analysis of the resin composite cylinders was performed for all groups to assess the presence of poorly polymerized and soft resin composites, as described in previous studies.^10,29,30^ Each pre-measured resin composite cylinder was subsequently immersed and sonicated (Gyromax 838, Mandel, Amerex Instruments, Inc., Concord, CA, USA) in 5 ml of Methyl-Ethyl-Ketone (MEK) (Sigma Aldrich, MilliporeSigma Co., Oakville, Ontario, Canada) for 2 hours. After sonication, the additional softened resin composite was scraped off. The samples were dried and the final lengths were measured and divided by two to yield post-MEK immersion DOC data.

### Statistical analysis

The microtensile bond strength results were analyzed using two-way ANOVA for each light-curing unit and material independently. A significance level of α=0.05 was set for both analyses. All data were submitted to normality tests (Shapiro-Wilk), followed by the Tukey’s post-hoc test for all pairwise multiple comparisons.The DOC scrape test results were analyzed using two separate approaches. First, for the pre-MEK immersion results, two-way ANOVA was independently performed for each light-curing unit, with the significance level set at α=0.05. The data was then subjected to normality tests (Shapiro-Wilk), followed by the Tukey’s post-hoc test for all pairwise multiple comparisons. Subsequently, each composite was independently analyzed by paired *t*-tests (two-tailed) with a significance level of α=0.05; this data was also subjected to normality tests (Shapiro-Wilk).The statistical analyses for both the microtensile bond strength and DOC scrape tests were performed using Sigma Plot 13.5 (Systat Software Inc., Systat Software Inc., CA, USA) software.

## Results

### Microtensile bond strength (µTBS)

The microtensile bond strength (µTBS) of the composites to dentin obtained using the PW curing unit (Table 1) demonstrated statistically higher bond strengths for TBF cured for 10 seconds than when it was cured for 20 seconds (P=0.001); however, no differences were observed for the other composites regarding curing times. Overall, for the PW curing unit, a 10-second curing time resulted in significantly lower bond strengths for FBF than the other two restorative composites. Conversely, for the 20-second curing time, higher bond strength values were observed for the conventional composite (T) (P=0.001). There was no significant effect of curing time and resin brand on the µTBS results of the samples cured with the MW light unit (Table 1). Each composite was isolated for further statistical analysis (Table 1). The conventional composite (T) showed statistically higher bond strengths when samples were light-cured with the PW curing unit, regardless of curing time (P=0.003). However, the corresponding bulk-fill restorative, TBF, showed a statistically significant interaction between curing unit and time, with higher bond strengths being observed within 10 seconds of curing with the PW curing unit (P<0.001). On the other hand, FBF did not appear to be affected by different curing protocols, including using different light-curing units or curing times (P=0.184).

**Table 1:** Microtensile bond strength of bulk-fill composites to dentin. Values are in MPa (SD)


LCU*	PW		MW	
Composite Resin	10 Seconds	20 seconds	10 Seconds	20 seconds
TBF Tetric Bulk Fill	54.8 (13.3) A,a,♦ n=75	46.2 (16.6) B,b,+ n=75	47.91 (15.1) A,a,+ n=75	51.1 (16.0) A,a,♦+ n=75
FBF Filtek Bulk Fill	46.75 (22.6) A,b,♦ n=78	49.6 (18.0) A,b,♦ n=86	51.3 (22.2) A,a,♦ n=82	51.7 (19.9) A,a,♦
T Tetric EvoCeram	54.0 (12.3) A,a,♦ n=71	55.8 (12.4) A,a,♦ n=76	51.3 (8.5) A,a,+ n=76	50.5 (12.1) A,a,+ n=78

Two independet Two-Way ANOVA statistical analysis were performed. First, to evaluate the effects of resin composite and curing time for each LCU individually, represeted by supercript letters. Second, to evaluate the effects of curing time and LCU for each resin composite indepently, represented by superscript symbols.

Capital letters compare curing times and lower case letters compare resin composites, for PW and MW separetly. Identical letters indicate no statistical significant difference between the values. Identical symbols indicate no statistical significant difference between the values, for each resin composite independently.

### Failure mode analysis

**Figure 2:**
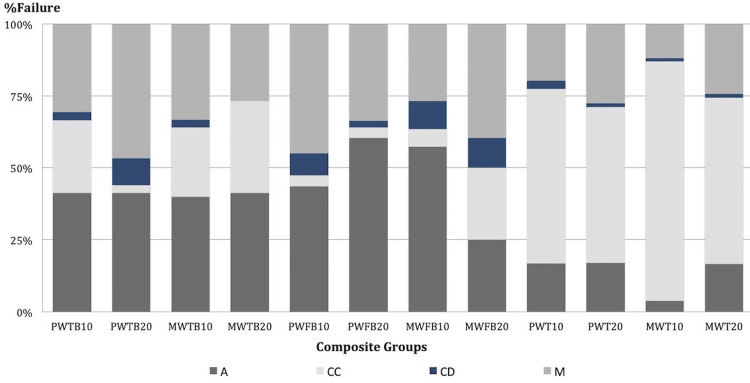
Failure mode distribution in percentage (%) for each experimental group. A: adhesive failures; CC: cohesive failures in composite; CD: cohesive failures in dentin; and M: mixed failures

**Figure 3:**
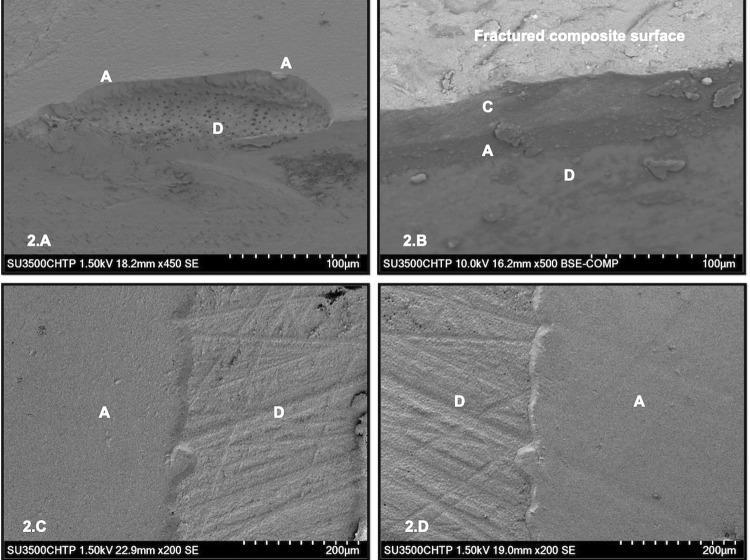
Scanning electron micrographs, representatives of the failure modes. A: Adhesive; D: Dentin; C: Composite. 2A: Mixed failure (M) seen in MW TBF cured for 20 seconds. It shows (A) and (D) surfaces involved in the same fracture. 2B: Cohesive failure in composite (CC) seen in MW T cured for 10 seconds. The image clearly shows the three distinct substrates, C, A, and D. 2C and 2D: mirror images showing a two-level fracture along the adhesive layer (A) observed in PW FTB cured for 20 seconds, partially de-bonding from the composite and partially from dentin

Figure 2 shows the overall failure mode. A higher percentage of adhesive and mixed failures were observed for both bulk-fill composites (TBF and FBF), regardless of the curing protocol applied. However, we observed a higher percentage of cohesive failures (50 to 75%) for the conventional composite (T), whereas mixed and adhesive failures represented a lower percentage. Cohesive failures in dentin represented only a small percentage. Figure 3 shows the representative scanning electron microscopy (SEM) images of each failure mode. The common pattern for cohesive failure in composites, most predominant for the conventional composite (T), are shown in Figure 3B. Cohesive failure in the composites occurred in most samples of that group, and in close proximity to the adhesive interface.

### Depth of cure (DOC)

The immediate, pre-MEK results of the scrape tests (Table 2) showed a significantly greater depth of cure for all resin composites light-cured for 20 seconds, regardless of the light-curing unit used (P<0.001). Both bulk-fill composites presented significantly greater DOC than the conventional composite, regardless of the curing time and unit (P<0.001). No statistically significant differences were observed between the two bulk-fill composites tested. Likewise, the post-MEK immersion scrape test results presented significantly higher DOC for all composites when cured with the PW curing unit for 20 seconds, and significantly higher DOC for both bulk-fill composites, regardless of the curing time. Table 3 shows the paired *t*-test analysis of pre-MEK and post-MEK immersion results for each composite. A statistically significant difference between pre- and post-MEK tests for all composites and all curing protocols was observed (P<0.001); however, all post-MEK groups presented a similar trend with a higher DOC for longer curing times and bulk-fill restorative materials.

**Table 2:** Scrape test results in mm (n=5)


LCU*	PW		MW	
Composite Resin	10 seconds	20 seconds	10 seconds	20 seconds

TBF Tetric EvoCeram Bulk Fill	3.02 (0.07)^B,a^	3.63 (0.18)^A,a^	2.84 (0.19)^B,a^	3.54 (0.06)^A,a^
FBF Filtek Bulk Fill	2.78 (0.12)^B,a^	3.53 (0.25)^A,a^	2.88 (0.05)^B,a^	3.44 (0.21)^A,a^
T Tetric EvoCeram	2.25 (0.22)^B,b^	2.74 (0.11)^A,b^	2.33 (0.08)^B,b^	2.72 (0.08)^A,b^

*Each light curing unit (LCU) was isolated for statistical analysis.

Superscript letters represent statistical differences. Capital letters compare curing times and lower-case letters compare resin composites.

**Table 3:** Scrape test results of each resin composite (n=5) analyzed independently (Paired *t*-test) pre- and *post*-MEK immersion. Data are represented in mm (SD)


Composite Resin*		TBF Tetric EvoCeram Bulk Fill		FBF Filtek Bulk Fill		T Tetric EvoCeram	
LCU	Curing Time	Before MEK immersion	After MEK immersion	Before MEK immersion	After MEK immersion	Before MEK immersion	After MEK immersion

PW	10s	3.02 (0.075)^a^	2.63 (0.19)^b^	2.78 (0.12)^a^	2.55 (0.21)^b^	2.25 (0.22)^a^	2.00 (0.2)^b^
	20s	3.63 (0.18)^a^	3.22 (0.17)^b^	3.53 (0.25)^a^	3.21 (0.19)^b^	2.74 (0.11)^a^	2.38 (0.14)^b^
MW	10s	2.84 (0.19)^a^	2.45 (0.2)^b^	2.88 (0.05)^a^	2.67 (0.02)^b^	2.33 (0.08)^a^	1.98 (0.04)^b^
	20s	3.54 (0.06)^a^	3.01 (0.07)^b^	3.44 (0.21)^a^	3.17 (0.198)^b^	2.72 (0.08)^a^	2.36 (0.05)^b^

*Each resin composite was isolated for statistical analysis. Superscript letters represent statistical differences.

## Discussion

The curing times selected for our research are consistent with those most commonly recommended for bulk-fill composites available on the market. Thus, the intent was to test both curing intervals (10 and 20 seconds) under similar radiant emittance^31^ (≅960 mW/cm^2^) for each light-curing unit. The first null hypothesis tested was partially rejected, as significantly lower µTBS values were observed for TBF, cured for 20 seconds with the PW curing unit (Table 1). One would expect higher µTBS values for TBF cured with the PW curing unit at 10 or 20 seconds due to complete compatibility of the adhesive-composite-curing unit system, which was supplied by the same manufacturer. However, lower µTBS values were observed for TBF cured for 20 seconds when compared with the 10-second curing time. Moreover, no differences were observed for TBF cured with either the PW or MW light-curing unit for the 20-second curing time (Table 1). Such unexpected findings could perhaps be attributed to a higher modulus of elasticity of the composite close to the adhesive interface due to enhanced curing of the corresponding resin composite (TBF), with the two-fold increase in its recommended curing time. As described in the literature, multiple factors can affect the stress distribution at the interface during tensile tests.^32^The stress along the bonded interface increases linearly with increasing elastic modulus of the bonded interface,^32,33^ which appears to justify our findings. This suggests that stiffer composite near the interface could generate higher stress during testing and cause failure at a lower force. In fact, fewer composite cohesive failures were observed (Figure 2) for this particular group (PW, TB, 20), which can support the argument. A potentially higher modulus at the interface could have resulted in increased interfacial stress; thus, significantly lowering the microtensile bond strengths. The increased DOC reported for this particular experimental group, described later in the discussion, also supports this statement.

The conventional composite (T), used as a control in our study, is recommended for placement in 2 mm increments to ensure optimal curing. In our research, however, the conventional composite was intentionally applied with the same parameters for all variables tested to fully understand the original research question. The conventional resin composite presented µTBS values comparable to both bulk-fill composites tested, even when light-cured for a shorter time (10 seconds) and when thickness was increased two-fold (4 mm increments). Thus, a sub-optimally cured conventional composite could potentially be present closer to the interface due to the combination of a shorter exposure time and thicker composite increments, attenuating the final energy delivered.^27^ Both factors combined present the rationale behind a more elastic-like behavior of the composite,^27^ especially closer to the adhesive interface, thus resulting in a higher apparent µTBS due to yielding of the resin composite before fracture. This appears supported by our findings in the failure mode analysis, where a high percentage of cohesive failures were observed for the conventional composite tested (Figure 2). Moreover, most of those aforementioned cohesive failures in the composite occurred close to the adhesive interface (Figure 3) and further from the light source, which can be associated with a reduced DOC (Table 2) and the resulting elastic behavior of the composite during the µTBS test.Furthermore, our specimens were stored for 24 hours, which is the most common procedure for all μTBS tests. However, this corresponds to a minimum termination time for maximum curing.^34^ Additional polymerization of the adhesive and composite build-ups could have resulted; thus eliminating major bond strength differences between the two curing times and among the resin composites tested. This has been demonstrated in previous studies, in which a significant increase in the degree of conversion and in the hardness of resin composites was observed after 24-hour storage.^20,22^

Van Ende, et al.^26^ (2013) studied the μTBS of bulk-fill resin composites compared to conventional composites in different C-factor cavities. No significant differences were found among the cavity configurations and the flat dentin surface for bulk-fill composites. However, the conventional composite presented significantly lower bond strength values when used for bulk-filling the cavities.^25^ In another recent study,^35^ the µTBS of either the bulk-fill or conventional resin composites showed higher µTBS values when 2 mm increments were used. Our study did not consider the cavity approach, but used flat dentin surfaces with the aim to eliminate potential confounding factors and exclusively evaluate the effects of curing protocols on the bond strength of the composites to dentin.

The resin composites evaluated in our study demonstrated different behavior under similar curing protocols, which parallels other studies on bulk-fill composites.^12,36^ Filtek Bulk-Fill composite (FBF) was the least influenced by the curing protocol with respect to µTBS values. However, for Tetric EvoCeram Bulk-Fill (TBF), using the polywave curing unit, resulted in overall improved bond strength values. This aligns with the manufacturer’s recommendations, since they clearly disclose the use of an alternative photoinitiator in the composite’s formulation. The polywave unit used in our study (Bluephase Style, Ivoclar Vivadent) is specifically recommended by the manufacturer for that composite and is fully compatible with Ivocerin^®^ (the alternative photoinitiator used). The absorption spectrum of Ivocerin^®^ falls within the blue-violet (390 nm) light range;^8^ thus leading to the conclusion that TBF could be effectively cured by the PW curing unit up to a depth of 4 mm with a minimum energy of 5.88 J/cm^2^.^12^

Failure mode analysis (Figure 2) indicated a higher percentage of adhesive and mixed failures for bulk-fill materials, which corresponds to the most common failure pattern in µTBS studies. Failure mode analysis for the conventional composite, on the other hand, presented the highest percentage of cohesive failures in the composites (up to 75%), which is a point for deeper consideration. Regarding the optical and SEM observations (Figure 3), most of these cohesive failures in the composite occurred very close to the adhesive interface, with a distinct layer of remaining conventional composite (≈ 0.5 mm) left adhered to the adhesive interface. The high percentage of cohesive composite failures in the conventional composite group could be related to the observations of Wakasa, et al.^33^ (1995) and Wasaka, Yamaki, Matsui^37^ (1995): the average stress at the interface was dependent upon the elasticity value ratio of the composite resin to the bonding area. These findings could explain the predominance of poorly cured composite near the interface for the conventional composite, since this material is not recommended for use in a bulk-filling technique. In addition, the nature of the experiment, with potentially undercured composite in some areas of the interface, may have had an impact in the variance of the data. Our DOC results support the findings from the μTBS tests, since the average DOC values for T (conventional composite) were around 2 mm, and even as low as 1.98 mm, for the 10-second curing time with the MW curing unit (Table 2). Moreover, these results are supported by previous investigations on the polymerization of restorative composites using 2 mm increments to allow adequate curing.^2,6,7,10^ Although we did not assess the degree of conversion or DOC of bonded samples, our results pertaining to the DOC could offer further evidence that explain the high percentage of cohesive failures in composites for the conventional composite (T) tested.

The second null hypothesis can be rejected. Both bulk-fill composites showed significantly greater DOC for both curing times and both light-curing units in pre- and post-MEK immersion. This finding aligns with the observations of Menees, et al.^17^ (2015), in which no significant differences in DOC were found with the use of the MW or PW curing units. Previous studies, however, have questioned the impact of the material composition and the corresponding light-curing units on the mechanical properties of the composite. One found a significant improvement in the degree of conversion and micro-hardness when a PW curing unit was used on a TPO-containing resin composite when compared with a MW curing unit.^18^ Nevertheless, methods are diverse and results must be carefully interpreted. All resin composites tested reflected greater DOC with the extended curing time (20 seconds). Other studies investigating polymerization properties of bulk-fill or conventional composites similarly observed that bulk-fill materials obtained sufficient polymerization properties at 4 mm depth, and increased curing time improved polymerization properties for both bulk-fill and conventional composites.^4,15,24^

The scraping method, as indicated by Rueggeberg, et al.^38^ (2009) correlates with flexural strength tests, which justifies its use in our study. In addition, the scraping method allows immediate measurements of the cured/uncured composite, thus minimizing the potential for additional curing that may occur during sample preparation and data collection for other mechanical tests (e.g. Vickers hardness or flexural strength). The scraping method can overestimate the DOC compared to the DOC determined by hardness profiles.^18^ However, the scraping method is a validated research tool^28^for direct comparison among materials, light-curing units and curing times. Moreover, the scrape test would permit further analysis of the specimens post-MEK immersion, allowing the assessment of the final remaining composite thickness after the removal of poorly cured composite. Recently, Daugherty, et al.^24^(2018) evaluated multiple bulk-fill composites for depth of cure using the scrape test method (ISO-4049) and depth of polymerization using FTIR. The results showed similar trends, with increased percentage values obtained upon increased mean irradiance (mW/cm^2^).

The paired *t*-test performed for all DOC samples showed a significant decrease in the remaining cured composite after Methyl-Ethyl-Ketone (MEK) immersion when compared to the pre-MEK immersion data (Table 3). These findings were consistent throughout the groups tested, regardless of curing time, light-curing unit, and resin composite tested. As previously reported, MEK or other solvents have been used to dissolve poorly cured composite;^29^ thus avoiding the overestimation of DOC for resin composites. To our knowledge, this is the first investigation to incorporate the use of solvents like MEK into the traditional ISO 4049 scrape test method. This is clear and valuable information to better refine the original scraping tests; nonetheless, the method still requires further exploration for ideal solvent and immersion time, along with their correlation with the degree of convergence and hardness. We could demonstrate that the 2-hour MEK immersion was capable of dissolving sub-optimally cured resin composite, which statistically impacted the post-MEK DOC results. Our results are consistent with previous results, which demonstrated higher DOC for bulk-fill materials when compared with their conventional counterparts.^21,22,30^

Finally, the DOC results of our study can directly support the findings on μTBS and failure modes. The average DOC values were lower than the 4 mm increment established for the μTBS tests. This could explain the high percentage of cohesive failures in the composite close to the interface due to the poorly polymerized composite observed in the conventional composite group. Therefore, the limited DOC (1.98 to 2.38 mm) of the conventional composite in post-MEK immersion analysis fully aligns with the failure mode findings. The poorly polymerized composite led to a higher percentage of composite cohesive failures using an experimental design that established a bulk-filling approach of 4 mm increments for the μTBS tests.

We selected two commercially available restorative bulk-fill composites, Filtek Bulk Fill and Tetric Bulk Fill. A newer version of the Filtek composite, Filtek One Bulk Fill, has recently been introduced. However, most recent studies report data related to the former formulation, compatible with the bulk fill composites used in our study.^5,6,39,40^ In addition, our study cannot expand on further discussion related to the potential interference of the two material composition, since they were not directly compared.

In short, our results raise concerns about the quality of polymerization of a resin composite applied in bulk and closer to the pulpal floor, especially in medium to deep cavities. Moreover, it is clear that μTBS test results are poor predictors of a well-cured composite (and vice versa), since other factors, such as material elasticity and its compliance under tensile stress^33,37^ may play a significant role, requiring further studies. Furthermore, our study was designed to assess immediate results only and did not propose aging in long-term storage at 37°C or termocycling; however, the information these tests could provide is relevant and should be considered for future studies in the field.

## CONCLUSION

Within the limitations of our study we can conclude that the curing modes did not result in differences in the μTBS of the composites to dentin, with the exception of lower bond strengths in the TBF composite cured with the PW unit for 20 seconds. The bond strength of conventional composites to dentin were comparable to the bulk-fill composites regardless of type of light unit and curing time. The DOC was greater for both bulk-fill composites evaluated, regardless of curing time and light-curing unit. Regarding the different curing times, the extended curing time (20 seconds) significantly increased the DOC for all composites and for both light-curing units. Finally, MEK immersion can significantly decrease the final DOC measurement for all resin composites.
